# Validity of the revised Ekblom Bak cycle ergometer test in adults

**DOI:** 10.1007/s00421-016-3412-0

**Published:** 2016-06-16

**Authors:** Frida Björkman, Elin Ekblom-Bak, Örjan Ekblom, Björn Ekblom

**Affiliations:** Åstrand Laboratory of Work Physiology, The Swedish School of Sport and Health Sciences, Box 5626, 114 86 Stockholm, Sweden

**Keywords:** Cardiorespiratory fitness, Estimation, Public health, Oxygen uptake, Submaximal test, Validity

## Abstract

**Purpose:**

To further develop the Ekblom Bak-test prediction equation for estimation of VO_2_max from submaximal cycle ergometry.

**Methods:**

The model group (117 men and 100 women, aged 48.3 ± 15.7 and 46.1 ± 16.8 years, VO_2_max 46.6 ± 11.1 and 40.4 ± 9.6 mL kg^−1^ min^−1^, respectively) and the cross-validation group (60 men and 55 women, aged 40.6 ± 17.1 and 41.6 ± 16.7 years, VO_2_max 49.0 ± 12.1 and 43.2 ± 8.9 mL min^−1^ kg^−1^, respectively) performed 4 min of cycling on a standard work rate (30 W) directly followed by 4 min on a higher work rate. Heart rate (HR) at each work rate was recorded. Thereafter, participants completed a graded maximal treadmill test for direct measurement of oxygen uptake. The new prediction equation was cross-validated and accuracy compared with the original Ekblom Bak equation as well as by the Åstrand test method.

**Results:**

The final sex-specific regression models included age, change in HR per-unit change in power (ΔHR/ΔPO), the difference in work rates (ΔPO), and HR at standard work rate as independent variables. The adjusted *R*^2^ for the final models were 0.86 in men and 0.83 in women. The coefficient of variation (CV) was 8.7 % and SEE 0.28 L min^−1^. The corresponding CV and SEE values for the EB-test_2012_ and the Åstrand tests were 10.9 and 18.1 % and 0.35 and 0.48 L min^−1^, respectively.

**Conclusion:**

The new EB-test prediction equation provides an easy administered and valid estimation of VO_2_max for a wide variety of ages (20–86 years) and fitness levels (19–76 mL kg^−1^ min^−1^).

## Introduction

Cardiorespiratory fitness, assessed as maximal oxygen consumption (VO_2_max), is a key factor in physical performance (Bassett and Howley 2000) and a strong independent predictor of health and longevity (Blair et al. 1989; Kodama et al. 2009). VO_2_max is determined during maximal physical effort by indirect calorimetry using special laboratory equipment. The methodology is time consuming, expensive, and dependent on physiological expertise. Furthermore, the VO_2_max measurements require a maximal effort from an individual, which is not always suitable in the general non-athlete population. Even if the maximal exercise test per se is a relatively safe procedure, there are also a number of contraindications that limits the possibility to evaluate VO_2_max in many settings (American Thoracic and American College of Chest 2003). To enable some evaluation of cardiorespiratory fitness in a population, in which the direct determination of VO_2_max is not possible, several tests, such as different step tests (Brouha et al. 1943; McArdle et al. 1972; Bennett et al. 2015), walk tests (Kline et al. 1987; Solway et al. 2001), treadmill (Swank et al. 2001), and cycle ergometer tests (Åstrand and Ryhming 1954; Legge and Bannister 1986; Golding et al. 1989; Hartung et al. 1993; Beekley et al. 2004), have been developed to estimate VO_2_max from submaximal performance.

In 2012, a submaximal cycle ergometer test (the original Ekblom-Bak test, EB-test_2012_) was presented, which estimates VO_2_max based on sex, age, and heart rate (HR) difference between a standard, low standard work rate, and a high individually chosen work rate (Ekblom-Bak et al. 2014). In a mixed population with regard to sex, age, and physical activity status, there was a strong correlation between estimated and actually measured VO_2_max, *r* = 0.91, with a corresponding coefficient of variation (CV) of 9.3 %. This was a significantly improved precision level compared with one of the most commonly used submaximal cycle ergometer tests and the Åstrand test (Åstrand 1960), reported by the authors to have a CV of 15 %. This is similar to other validation reports (Jessup et al. 1977; Siconolfi et al. 1982; Ekblom et al. 2007) and to the values found in our study population in the 2012 publication (*r* = 0.68 and CV 18.1 %) (Ekblom-Bak et al. 2014).

Although the relatively high precision of estimating VO_2_max by the EB-test_2012_, there was a strong, significant correlation between the error of measured and estimated VO_2_max and absolute VO_2_max level (spearman *ρ* = 0.42 in the total population, *ρ* = 0.51 in women, and *ρ* = 0.81 in men). This means that individuals with high VO_2_max were underestimated and individuals with low VO_2_max overestimated with the EB-test_2012_. Moreover, during the development of the EB-test_2012_ prediction equation, we found that the size of the study population limited the possibility of sex-specific equations, something that probably would enhance the prediction equation as sex was included as independent variable in the prediction equation. In addition, by including a sample having a greater range of VO_2_max (currently for the EB-test_2012_ prediction equation 1.56–3.73 L min^−1^ in women and 2.75–4.49 L min^−1^ in men) and age (currently 21–65 years), the test would be applicable on a greater proportion of the general population.

Therefore, in the present paper, we aimed to further develop the EB-test_2012_ prediction equation by including additional participants to the study population on which the 2012 prediction equation was developed. This enabled us to develop sex-specific prediction equations and to expand the valid age and VO_2_max range for the test. By identifying individual characteristics and physiological responses associated to VO_2_max, we also aimed to reduce the estimation bias seen with high VO_2_max level in the original test. The new prediction equation (EB-test_new_) was then validated internally in the model group and in an external cross-validation sample and was also compared with the Åstrand and the EB-test_2012_.

## Materials and methods

### Participants

After public announcement and word-of-mouth in the region of Stockholm, Sweden, we included 74 additional participants to the 2012 model group (Ekblom-Bak et al. 2014). This additional sample consisted of 52 men and 22 women, mean age 58.8 (20–86) years, with a mean VO_2_ max of 3.10 (1.33–5.97) L min^−1^ and 39.2 (18.0–76.4) mL kg^−1^ min^−1^. Inclusion criteria for this group were an age, and VO_2_max mainly identified to be outside the valid range for the 2012 prediction equation (Ekblom-Bak et al. 2014). These participants were thereafter pooled to the model group of the 2012 prediction equation. The pooled sample was used to create the new prediction equation for the EB-test. The same recruitment method as for the participants to the model group was applied for the cross-validation sample. Recruitment of the latter sample consisted of a mixture of men and women of different ages and fitness levels, to enable cross validation in both the total sample and in subgroups. Inclusion criteria were men and women above 20 years of age, with no known diseases or disabilities. The characteristic of both the new model group (*n* = 217) as well as the cross-validation sample (*n* = 115) is presented in Table 1.

**Table 1:** Characteristics of the study samples (means ± standard deviations)

	Model group for the EB-test_new_ equation		Cross-validation group for the EB-test_new_ equation		Comparison group^a^	
	Men (*n* = 117)	Women (*n* = 100)	Men (*n* = 60)	Women (*n* = 55)	Men (*n* = 27)	Women (*n* = 44)
Age (years)	48.3 (15.7)	46.1 (16.8)	40.6 (17.1)	41.6 (16.7)	36.0 (12.8)	36.9 (13.7)
Height (cm)	180.4 (6.9)	166.2 (6.2)	180.4 (6.0)	167.3 (6.9)	179.0 (5.6)	167.2 (7.2)
Body Mass (kg)	80.7 (9.0)	63.7 (8.5)	82.5 (15.4)	63.9 (8.8)	81.8 (17.2)	63.5 (7.9)
VO_2_max (L min^−1^)	3.73 (0.86)	2.55 (0.58)	3.95 (0.89)	2.75 (0.59)	3.70 (0.55)	2.85 (0.53)
VO_2_max (mL kg^−1^ min^−1^)	46.6 (11.1)	40.4 (9.6)	49.0 (12.1)	43.2 (8.9)	46.4 (8.4)	45.1 (7.9)
HR_max_ (beats min^−1^)	178 (17)	179 (12)	185 (15)	182 (12)	192 (11)	185 (11)

^a^Including those participants in the cross-validation group with a valid EB-test_2012_ and Åstrand test

All participants were free from all the types of diseases that limit the physical work capacity and stated themselves as healthy on the test day. Furthermore, they were not taking any medications that could influence the relationship between HR and VO_2_. Exclusion criteria were smoking, snuff use, and medication with beta blockers or asthmatic medicine. Participants visited the test laboratory on one occasion to perform the submaximal EB-test and a maximal treadmill test to assess actual VO_2_max. Before the visit, the participants were asked to refrain from smoking and vigorous physical activity the day before and on the test day, and to not consume a heavy meal less than 3 h before the test. All participants were fully informed about the details of the study and provided written consent.

### Submaximal and maximal test

All tests were performed in climate-controlled laboratory environment. When arriving to the test centre, body mass (measured while wearing in light-weight clothes to the nearest 0.1 kg) and height (to the nearest 0.1 cm) were measured. The participants were informed about the test procedure and equipped with a HR monitor (Polar Electro, Kempele, Finland). After individual adjustments of the seat and handlebar of the cycle ergometer and an introduction of the Borg´s scale of perceived exertion (RPE) (Borg 1970), the participant performed an EB-test according to the original 2012 test procedure (Ekblom-Bak et al. 2014). The test was performed on a mechanically braked cycle ergometer (Monark model 828E, Varberg, Sweden). Test procedure included 4 min of cycling on a standard and low work rate of 0.5 kilopond (kp) with a pedal frequency of 60 rpm (≈30 W when 1 W = 6.116 kpm/min), directly followed by 4 min of cycling on a higher individually chosen work rate (aiming at a RPE of ≈14 on the Borg scale). Mean steady-state HR during the last minute on the low and high work rates, respectively, was recorded by taking the mean of the observed HR at 3:15, 3:30, 3:45, and 4:00 min at each work rate. In addition, VO_2_max was also estimated by the Åstrand test method by applying the work rate and HR of the high work rate to the Åstrand nomogram (Åstrand and Ryhming 1954) and associated age-correction factors (Åstrand 1960). The same way of obtaining Åstrand test results from the EB-test procedure was used in the original publication of the first EB-test prediction equation, and is further described and discussed in the previous article (Ekblom-Bak et al. 2014). Direct measurement of VO_2_ during the submaximal cycle test was conducted in a subsample (*n* = 110) in the model group, using a computerised metabolic system (Jaeger Oxycon pro, Hoechberg, Germany) connected to a face mask worn by the participant. Before each test, ambient temperature, humidity, and barometric pressure were measured with built-in automatic procedures and a handheld instrument (HygroPalm, Rotronic, Bassersdorf, Schweiz). Gas analyzers and inspiratory flowmeter were calibrated with the metabolic system’s built-in automatic procedures, where high-precision calibration gases (15.00 ± 0.01 % O_2_ and 6.00 ± 0.01 % CO_2_, Air Liquid, Kungsängen, Sweden), and ambient indoor air was used for the gas analyses.

After a short rest, a 5 min warm-up on the treadmill preceded a graded maximal treadmill test to measure VO_2_max. The individually designed protocol for the VO_2_max test started off at 1° incline and a velocity corresponding to approximately 60–65 % of the participant’s estimated VO_2_max (usually the speed that the participant felt comfortable with during the warm-up). The speed increased 1 km/h during the first 3 to 4 min of the test, and thereafter, there was an increase in incline with +1° every minute until voluntary exhaustion. For some of the well-trained participants, running to an incline of 5°–6°, there was an additional increase in speed (+1 km h^−1^ per minute) to avoid too steep inclination on the treadmill. Direct measurements of VO_2_ were obtained during the test with the same computerised system as mentioned above (Jaeger Oxycon pro). Criteria for acceptance of the VO_2_max measurement were levelling off of VO_2_ despite an increase in speed or incline, a respiratory exchange ratio >1.1, RPE above 16, work time above 6 min, supported by a maximal HR within ±15 beats min^−1^ (bpm) from age-predicted maximal HR (ref Åstrand Rodahl). A test was accepted as VO_2_max when a minimum of three out of the five criteria was achieved. In the model group, nine participants were tested but later excluded due to non-fulfilling the requirements for acceptance of test (five participants failed the VO_2_max test and four participants had non-valid EB test). The corresponding values in the cross-validation group were four excluded participants in total, two with non-valid VO_2_max test and two with non-valid EB-test.

VO_2_max (L min^−1^) and maximal HR (bpm) were recorded into 30 and 5 s epochs, respectively. We have previously shown that there is no mean difference and a small variation (CV: 2.7 %) between test–retest of VO_2_max according to the above procedure in a mixed population (Ekblom-Bak et al. 2014), indicating no need for a second VO_2_max test on a separate test day to verify the first accepted measurement.

### Development of the new EB-test prediction equation

In the EB-test_2012_ prediction equation, VO_2_max is predicted by entering sex (women = 0, men = 1) and age (years), and the difference in HR between the high and low work rate (ΔHR) divided by the difference in work rate between the high and low work rate (ΔPO) into the equation VO_2_max = 4.98196 − 2.88618 (ΔHR/ΔPO) + 0.65015 (sex) − 0.01712 (age). For the development of ΔHR/ΔPO, see the paper of the EB-test_2012_ (Ekblom-Bak et al. 2014). In line with the development of the EB-test_2012_ equation, but now based on the extended model group population, linear regression modelling was used to develop the EB-test_new_ prediction equation. Apart from performing separate prediction equations for men and women, the natural logarithm of VO_2_max (ln VO_2_max) was used for a better fit and for avoiding extrapolation to infinity in both ends of the regression. Since both the ΔPO variable (representing the high work rate) and the steady-state HR obtained on the low, the standard work rate was identified as highly associated with VO_2_max in both men and women in the EB-test_2012_ equation (for ΔPO, *r* = 0.76 in women and 0.79 in men; for HR at the standard work rate, *r* = −0.57 in women and *r* = −0.26 in men; *p* < 0.001 for all); we chose to include these as separate variables in the new model. The model construction is described in detail in the statistical analysis section below.

### Validation, cross validation, and comparison groups

The EB-test_new_ prediction equation presented in the results section was internally validated in the model group as well as in the external cross-validation sample. Moreover, the validity and precision of the estimated VO_2_max by the EB-test_new_ was also compared with both the EB-test_2012_ and estimated VO_2_max derived from the Åstrand test method. This group is called the comparison group and consisted of only participants from the cross-validation group. Since both the EB-test_2012_ and the Åstrand test method have narrower valid age and VO_2_max ranges compared with the new prediction equation, only those fulfilling the prerequisites (i.e., participants aged 20–65 years with an HR on the high work rate applicable to the Åstrand nomogram, namely, 120–170 bpm) for all three tests were included in these comparison analyses (see Table 1 for the characteristics of participants in the validation group, cross-validation group, and comparison group).

VO_2_max estimated by the EB-test_2012_ was obtained by entering the corresponding values for each participant into the equation presented above. Results are also reported for quartiles in fitness level for absolute (L min^−1^) as well as relative (mL kg^−1^ min^−1^) VO_2_max.

### Statistical analysis

A multiple linear regression with the forward method (probability of *F* = 0.05 for entry, and 0.10 for removal) was used, identifying age, ΔHR/ΔPO, ΔPO, and HR at the low standard work rate to be included in the final model as independent predictors of ln VO_2_max. Since we found significant gender-HR and gender-∆HR/∆PO interaction effects (both *p* < 0.001) for the prediction of ln VO_2_max, regressions were performed for men and women separately. The final model was checked for homoscedasticity and equal variance. Tolerance values >0.2 for all independent variables indicated low probability of multicollinearity. The 95 % confidence intervals (95 % CI) for the regression coefficients are given. Paired *t* tests were used to study the difference between measured and estimated VO_2_max. Bland–Altman plots, including limits of agreement (LoA), were produced for the cross-validation sample. The coefficient of variation variable (Tables 2, 3, 4) was calculated by dividing the standard deviation (SD) of the difference between measured and estimated VO_2_max with the mean of the measured and estimated VO_2_max. The adjusted *R*^2^ and standard errors of the estimate (SEE) were obtained by linear regression, entering measured VO_2_max as the dependent variable and calculated VO_2_max as the independent variable. Two-sided statistical significance was set to *p* < 0.05 for all analyses. The SPSS statistical software version 21.0 (SPSS Inc., Chicago, IL, USA) was used for the statistical analyses.

**Table 2:** Validity of the EB-test_new_ equation (in the model group)

	Measured VO_2_max (L min^−1^)	Estimated VO_2_max (L min^−1^)	Difference measured vs estimated VO_2_max (L min^−1^)	Coefficient of variation (%)	*R* ^2^ adjusted	SEE (L min^−1^)
	Mean (SD)	Mean (SD)	Mean (95 % CI)			
All (*n* = 217)	3.18 (0.95)	3.17 (0.92)	−0.01 (−0.05 to 0.03)	8.7	0.91	0.28
Men (*n* = 117)	3.73 (0.86)	3.72 (0.83)	−0.01 (−0.07 to 0.05)	8.4	0.87	0.31
Women (*n* = 100)	2.55 (0.58)	2.53 (0.52)	−0.01 (−0.06 to 0.03)	9.2	0.84	0.24
Age						
<35 (*n* = 59)	3.57 (0.91)	3.63 (0.91)	0.06 (−0.02 to 0.13)	7.9	0.90	0.28
35–49 (*n* = 61)	3.65 (0.86)	3.59 (0.83)	−0.06 (−0.12 to 0.01)	6.8	0.92	0.25
50–64 (*n* = 49)	3.03 (0.71)	2.94 (0.64)	−0.09 (−0.18 to −0.01)	10.3	0.81	0.31
≥65 (*n* = 48)	2.27 (0.55)	2.32 (0.51)	0.05 (−0.03 to 0.12)	10.9	0.80	0.25
Absolute VO_2_max level (according to L min^−1^)						
Q1 (*n* = 55)	2.24 (0.53)	2.34 (0.53)	0.10 (0.04 to 0.16)	9.9	0.82	0.22
Q2 (*n* = 54)	2.95 (0.58)	2.95 (0.60)	−0.00 (−0.08 to 0.09)	11.0	0.72	0.31
Q3 (*n* = 54)	3.47 (0.66)	3.46 (0.73)	−0.01 (−0.07 to 0.05)	6.5	0.91	0.20
Q4 (*n* = 54)	4.09 (0.84)	3.95 (0.87)	−0.14 (−0.21 to −0.06)	6.9	0.90	0.27
Relative VO_2_max level (according to mL kg^−1^ min^−1^)						
Q1 (*n* = 54)	2.27 (0.57)	2.36 (0.57)	0.09 (0.02 to 0.16)	10.5	0.82	0.24
Q2 (*n* = 54)	3.02 (0.62)	2.96 (0.63)	−0.06 (−0.14 to 0.01)	9.3	0.81	0.27
Q3 (*n* = 55)	3.48 (0.78)	3.44 (0.73)	−0.03 (−0.12 to 0.05)	8.8	0.85	0.31
Q4 (*n* = 54)	3.96 (0.87)	3.92 (0.90)	−0.04 (−0.11 to 0.03)	6.6	0.92	0.25

All measured and estimated values are in expressed as L min^−1^

Quartiles (L min^−1^), women; <2.14, 2.14–2.59, 2.59–2.93, >2.93; Men; <3.07, 3.07–3.75, 3.75–4.33, >4.33

Quartiles (mL kg^−1^ min^−1^), women; <32.5, 32.5–40.2, 40.2–47.4, >47.4; men; <37.5, 37.5–47.6, 47.6–54.8, >54.8

*SD* standard deviation, *SEE* standard error of the estimate, *Q* quartile

**Table 3:** Cross validation of the EB-test_new_ equation (in the external cross-validation group)

	Measured VO_2_max (L min^−1^)	Estimated VO_2_max (L min^−1^)	Difference measured vs estimated VO_2_max (L min^−1^)	Coefficient of variation (%)	*R* ^2^ adjusted	SEE (L min^−1^)
	Mean (SD)	Mean (SD)	Mean (95 % CI)			
All (*n* = 115)	3.37 (0.97)	3.39 (1.02)	0.02 (−0.04 to 0.08)	9.4	0.90	0.30
Men (*n* = 60)	3.95 (0.89)	4.06 (0.89)	0.11 (0.02 to 0.20)	8.3	0.86	0.33
Women (*n* = 55)	2.75 (0.59)	2.66 (0.54)	−0.09 (−0.16 to −0.01)	10.0	0.79	0.27
Age						
<35 (*n* = 55)	3.75 (0.77)	3.77 (0.90)	0.01 (−0.08 to 0.11)	9.1	0.86	0.29
35–49 (*n* = 28)	3.53 (1.07)	3.49 (1.07)	−0.04 (−0.15 to 0.08)	8.5	0.92	0.30
50–64 (*n* = 15)	2.87 (0.81)	2.95 (0.89)	0.08 (−0.11 to 0.27)	11.8	0.84	0.33
≥65 (*n* = 17)	2.34 (0.49)	2.39 (0.56)	0.06 (−0.07 to 0.19)	10.9	0.77	0.23
Absolute VO_2_max level (according to L min^−1^)						
Q1 (*n* = 24)	2.28 (0.47)	2.44 (0.59)	0.16 (0.03 to 0.29)	12.8	0.73	0.24
Q2 (*n* = 21)	2.96 (0.51)	3.02 (0.71)	0.06 (−0.07 to 0.18)	9.3	0.90	0.16
Q3 (*n* = 23)	3.41 (0.67)	3.38 (0.80)	−0.03 (−0.14 to 0.09)	8.0	0.89	0.22
Q4 (*n* = 47)	4.10 (0.81)	4.05 (0.96)	−0.05 (−0.15 to 0.05)	8.5	0.88	0.29
Relative VO_2_max level (according to mL kg^−1^ min^−1^)						
Q1 (*n* = 23)	2.58 (0.70)	2.68 (0.66)	0.10 (−0.01 to 0.21)	9.3	0.87	0.25
Q2 (*n* = 19)	3.00 (0.82)	3.02 (0.91)	0.03 (−0.10 to 0.15)	8.8	0.91	0.24
Q3 (*n* = 31)	3.32 (0.80)	3.36 (0.93)	0.04 (−0.10 to 0.19)	11.9	0.82	0.34
Q4 (*n* = 42)	4.02 (0.86)	3.97 (0.99)	−0.05 (−0.15 to 0.04)	7.7	0.91	0.26

All measured and estimated values are in expressed as L min^−1^

Quartiles (L min^−1^), women; <2.14, 2.14–2.59, 2.59–2.93, >2.93; men; <3.07, 3.07–3.75, 3.75–4.33, >4.33

Quartiles (mL kg^−1^ min^−1^), women; <32.5, 32.5–40.2, 40.2–47.4, >47.4; men; <37.5, 37.5–47.6, 47.6–54.8, >54.8

*SD* standard deviation, *SEE* standard error of the estimate, *Q* quartile

**Table 4:** Comparison of the three tests (in the comparison group)

	Measured VO_2_max (L min^−1^)	Estimated VO_2_max (L min^−1^)			Difference measured vs. estimated VO_2_max (L min^−1^)		
		EB-test_new_	EB-test_2012_	Åstrand	EB-test_new_	EB-test_2012_	Åstrand
	Mean (SD)	Mean (SD)	Mean (SD)	Mean (SD)	Mean (95 % CI)	Mean (95 % CI)	Mean (95 % CI)
All (*n* = 71)	3.17 (0.67)	3.14 (0.71)	3.30 (0.66)	3.10 (0.79)	−0.03 (−0.10 to 0.04)	0.12 (0.04 to 0.21)	−0.07 (−0.21 to 0.06)
Men (*n* = 27)	3.70 (0.55)	3.79 (0.53)	3.89 (0.33)	3.29 (0.75)	0.09 (−0.03 to 0.21)	0.20 (0.05 to 0.34)	−0.41 (−0.61 to −0.20)
Women (*n* = 44)	2.85 (0.53)	2.74 (0.47)	2.93 (0.54)	2.98 (0.80)	−0.11 (−0.19 to −0.03)	0.08 (−0.02 to 0.18)	0.13 (−0.02 to 0.28)
Age							
<35 (*n* = 41)	3.41 (0.50)	3.37 (0.56)	3.54 (0.43)	3.37 (0.66)	−0.04 (−0.13 to 0.06)	0.14 (0.03 to 0.24)	−0.04 (−0.23 to 0.14)
35–49 (*n* = 17)	2.97 (0.67)	2.91 (0.72)	3.06 (0.56)	2.81 (0.80)	−0.06 (−0.21 to 0.09)	0.10 (−0.04 to 0.24)	−0.15 (−0.48 to 0.17)
50–64 (*n* = 12)	2.75 (0.86)	2.77 (0.89)	2.86 (1.01)	2.69 (0.85)	0.02 (−0.19 to 0.23)	0.11 (−0.23 to 0.45)	−0.06 (−0.36 to 0.24)
Absolute VO_2_max level (according to L min^−1^)							
Q1 (*n* = 10)	2.19 (0.43)	2.36 (0.55)	2.52 (0.74)	2.24 (0.55)	0.16 (−0.05 to 0.37)	0.32 (0.00 to 0.64)	0.05 (−0.33 to 0.42)
Q2 (*n* = 15)	3.00 (0.47)	3.08 (0.68)	3.37 (0.62)	2.67 (0.62)	0.08 (−0.08 to 0.24)	0.37 (0.21 to 0.53)	−0.33 (−0.62 to −0.05)
Q3 (*n* = 23)	3.41 (0.67)	3.38 (0.80)	3.49 (0.69)	3.22 (0.67)	−0.03 (−0.14 to 0.09)	0.08 (−0.07 to 0.23)	−0.18 (−0.39 to 0.02)
Q4 (*n* = 23)	3.47 (0.41)	3.27 (0.44)	3.40 (0.35)	3.63 (0.62)	−0.20 (−0.31 to −0.10)	−0.08 (−0.16 to 0.01)	0.15 (−0.12 to 0.43)
Relative VO_2_max level (according to mL kg^−1^ min^−1^)							
Q1 (*n* = 11)	2.84 (0.79)	2.93 (0.72)	3.25 (0.77)	2.61 (0.55)	0.09 (−0.07 to 0.25)	0.40 (0.25 to 0.56)	−0.24 (−0.73 to 0.25)
Q2 (*n* = 12)	2.94 (0.82)	2.98 (0.89)	3.08 (0.96)	2.84 (0.87)	0.03 (−0.14 to 0.21)	0.14 (−0.10 to 0.38)	−0.10 (−0.37 to 0.16)
Q3 (*n* = 25)	3.21 (0.62)	3.20 (0.79)	3.34 (0.65)	3.01 (0.75)	−0.01 (−0.16 to 0.14)	0.13 (−0.02 to 0.28)	−0.19 (−0.42 to 0.03)
Q4 (*n* = 23)	3.41 (0.50)	3.25 (0.50)	3.38 (0.40)	3.56 (0.67)	−0.15 (−0.25 to −0.06)	−0.03 (−0.16 to 0.11)	0.15 (−0.08 to 0.39)

	Coefficient of variation			R^2^ adjusted			SEE		
	EB-test_new_ (%)	EB-test_2012_ (%)	Åstrand (%)	EB-test_new_	EB-test_2012_	Åstrand	EB-test_new_	EB-test_2012_	Åstrand
All (*n* = 71)	9.4	10.9	18.1	0.83	0.74	0.50	0.28	0.35	0.48
Men (*n* = 27)	8.4	9.7	14.8	0.68	0.56	0.50	0.31	0.36	0.39
Women (*n* = 44)	9.3	11.8	17.2	0.75	0.62	0.61	0.26	0.32	0.33
Age									
<35 (*n* = 41)	8.8	9.5	17.3	0.71	0.57	0.25	0.27	0.33	0.44
35–49 (*n* = 17)	9.9	9.1	21.7	0.83	0.82	0.38	0.28	0.28	0.52
50–64 (*n* = 12)	11.9	19.0	17.2	0.85	0.70	0.69	0.33	0.47	0.47
Absolute VO_2_max level (according to L min^−1^)									
Q1 (*n* = 10)	12.8	18.8	23.9	0.69	0.67	0.08	0.24	0.24	0.41
Q2 (*n* = 15)	9.7	9.2	18.2	0.86	0.78	0.29	0.17	0.22	0.39
Q3 (*n* = 23)	8.0	10.1	14.5	0.89	0.75	0.53	0.22	0.34	0.46
Q4 (*n* = 23)	7.3	5.7	17.7	0.69	0.76	0.06	0.23	0.20	0.40
Relative VO_2_max level (according to mL kg^−1^ min^−1^)									
Q1 (*n* = 11)	8.2	7.8	26.9	0.91	0.90	0.12	0.25	0.25	0.75
Q2 (*n* = 12)	9.3	12.5	14.6	0.90	0.84	0.75	0.27	0.33	0.41
Q3 (*n* = 25)	11.3	10.9	17.4	0.79	0.70	0.47	0.28	0.34	0.45
Q4 (*n* = 23)	6.6	9.2	15.5	0.81	0.59	0.34	0.22	0.32	0.41

All measured and estimated values are in expressed as L min^−1^

Quartiles (L min^−1^), women; <2.14, 2.14–2.59, 2.59–2.93, >2.93; men; <3.07, 3.07–3.75, 3.75–4.33, >4.33

Quartiles (mL kg^−1^ min^−1^), women; <32.5, 32.5–40.2, 40.2–47.4, >47.4; men; <37.5, 37.5–47.6, 47.6–54.8, >54.8

*SD* standard deviation, *SEE* standard error of the estimate, *Q* quartile

## Results

Subject characteristics in the model group, cross-validation group, and the comparison groups are shown in Table 1. The age and VO_2_max ranges for the participants in the model group were 21–86 years and 1.33–3.94 L min^−1^ (18.9–61.9 mL kg^−1^ min^−1^) in women, respectively, and 20–84 years and 1.67–5.97 L min^−1^ (23.5–76.4 mL kg^−1^ min^−1^) in men, respectively.

### New equation based on data from the model group

The final sex-specific regression models (with 95 % CI for the independent variables) for EB-test_new_ were

Men: ln VO_2_max = 2.04900 (95 % CI 1.83517–2.26282) − 0.00858 (95 % CI −0.00987 to 0.00728) (age) − 0.90742 (95 % CI −1.11676 to −0.69808) (ΔHR/ΔPO) + 0.00178 (95 % CI 0.00127–0.00228) (ΔPO) − 0.00290 (95 % CI −0.00438 to −0.00141) (HR at standard work rate).

Women: ln VO_2_max = 1.84390 (95 % CI 1.53151–2.15628) − 0.00673 (95 % CI −0.00812 to 0.00534) (age) − 0.62578 (95 % CI −0.81368 to −0.43789) (ΔHR/ΔPO) + 0.00175 (95 % CI 0.00056–0.00295) (ΔPO) − 0.00471 (95 % CI −0.00674 to −0.00268) (HR at standard work rate).

After entering the corresponding values into the equation, VO_2_max (in L min^−1^) was estimated by putting in the obtained value (*x*) as an exponent in the natural logarithm. *R*^2^ adjusted for the final models were 0.86 in men and 0.83 in women.

Table 2 presents the validity of the new equation in the model group. The systematic error (the difference between measured and estimated VO_2_max) was assessed in the full sample, as well as in subgroups for age and fitness level (presented as the quartiles for relative as well as absolute VO_2_max). The systematic error in the different subgroups ranges from an underestimation at the most of 0.14 L min^−1^ to an overestimation of 0.10 L min^−1^. The coefficient of variation was 8.7 % in the full sample, ranging between 6.5 and 11.0 % in the subgroups. In addition, the explained variance of the measured VO_2_max by the estimated value was 91 % in the full sample, and the SEE values were 0.28 L min^−1^ for the full sample and ranging between 0.20 and 0.31 L min^−1^ in the subgroups.

The differences between measured and estimated VO_2_max by the EB-test_new_ equation in the model group were not associated with maximal HR in women (Spearman *ρ* = 0.10, *p* = 0.33) or men (*ρ* = 0.14, *p* = 0.13), and not with deviation for age-predicted maximal HR (*ρ* = −0.17, *p* = 0.08) in women, but in men (*ρ* = −0.33, *p* < 0.001). In both women and men, the differences were associated with VO_2_max level, *ρ* = 0.40, *p* < 0.001, and *ρ* = 0.25, *p* = 0.006, respectively.

### Cross validation

**Fig. 1:**
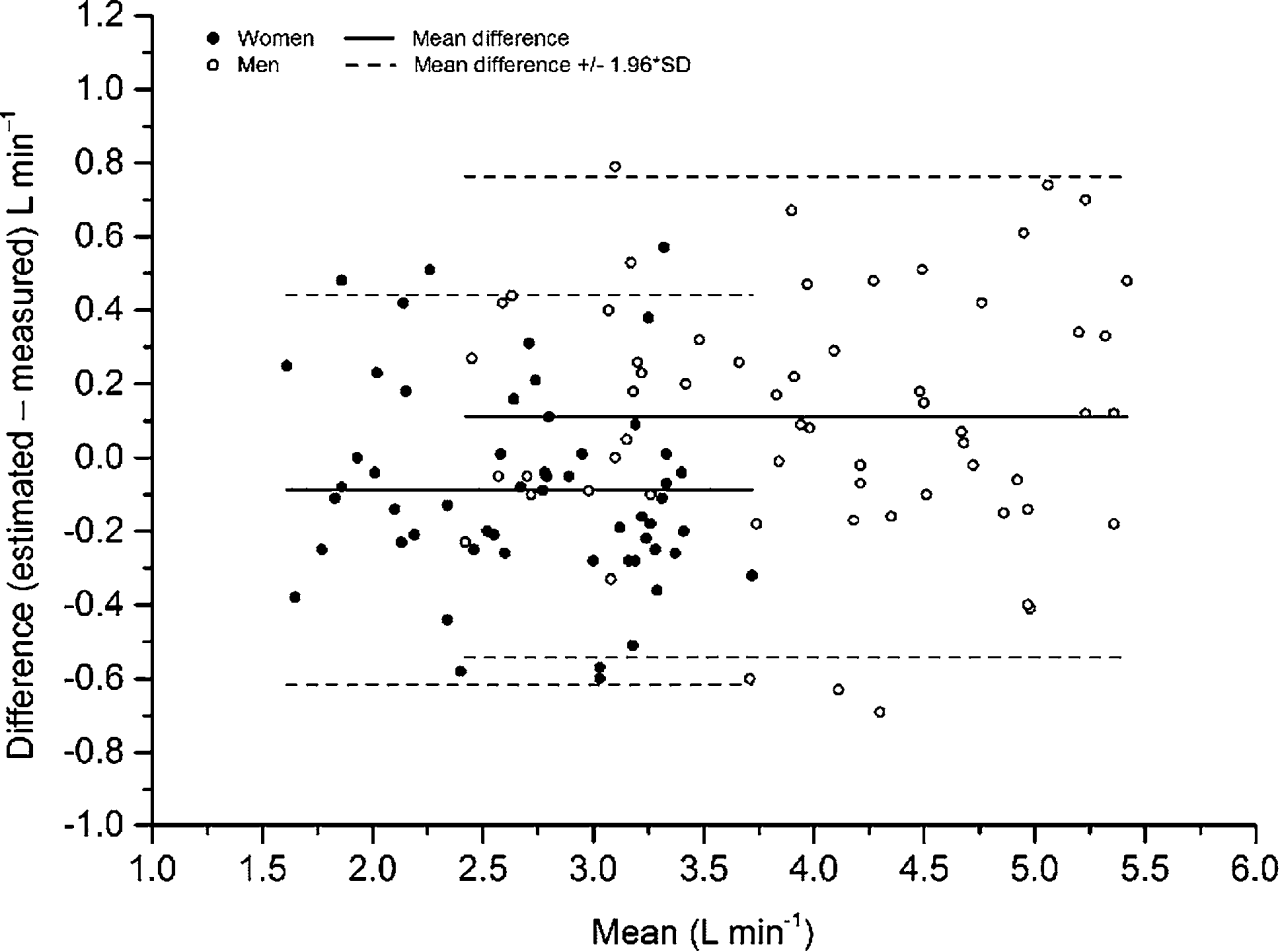
Bland Altman plot, including limits of agreement, for estimated and measured VO_2_max (L min^−1^) in the cross-validation group. *Filled dots* women. *Transparent dots* men. *Black line* mean difference. *Dashed line* ±1.96 × SD

**Fig. 2:**
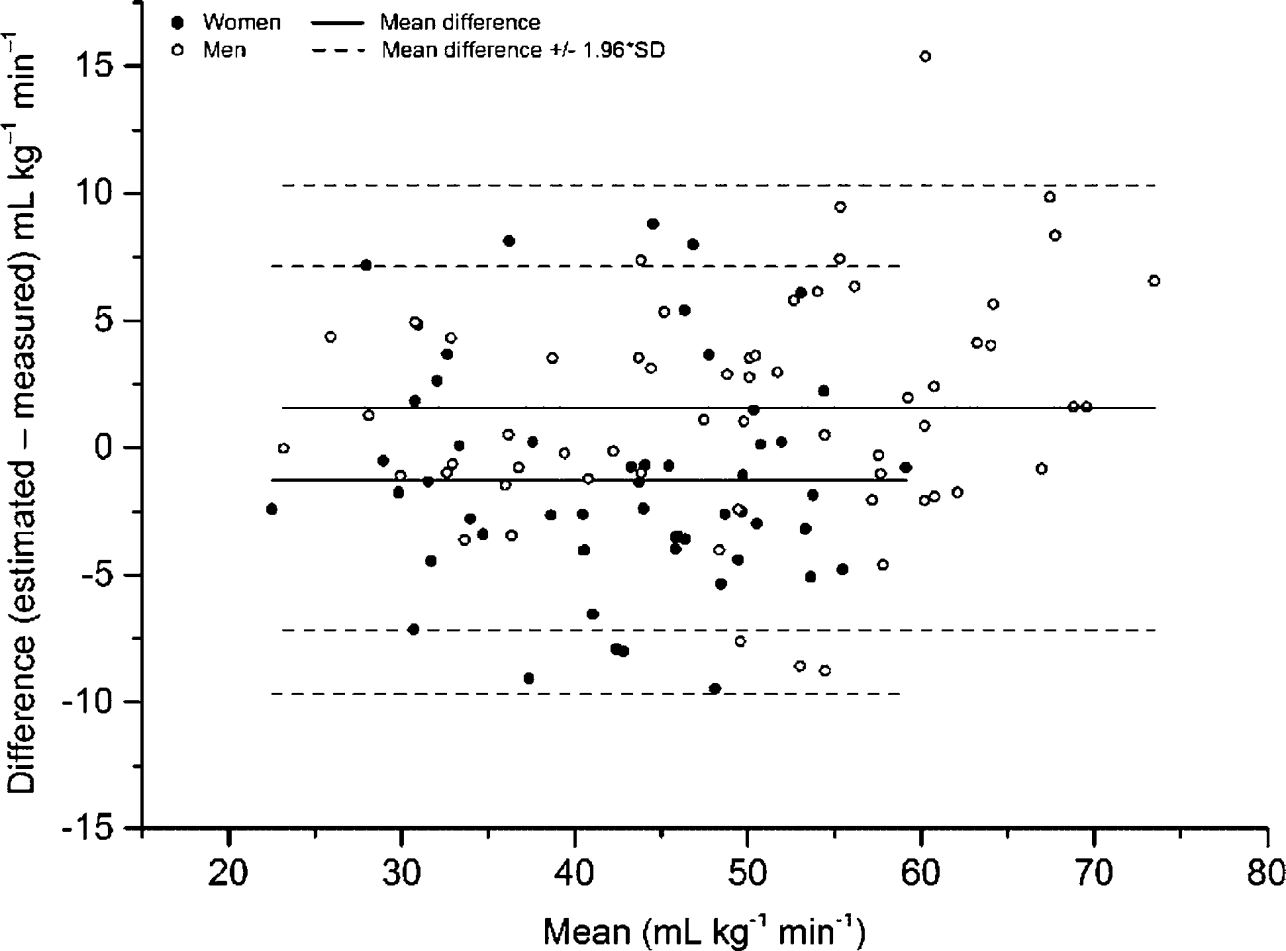
Bland Altman plot, including limits of agreement, for estimated and measured VO_2_max (mL kg^−1^ min^−1^) in the cross-validation group. *Filled dots* women. *Transparent dots* men. *Black line* mean difference. *Dashed line* ±1.96 × SD

In the cross-validation sample, the estimation of VO_2_max by the EB-test_new_ equation was analysed in the full sample and thereafter stratified into the same subgroups as the model group. Data were found to be homoscedastic. Results are shown in Table 3. The cross-validation analyses showed similar results as in the internal validation sample, with a non-significant mean systematic difference of 0.02 (95 % CI −0.04 to 0.08) and a similar variation, 9.4 %, in the full sample, ranging from −0.09 to 0.16 L min^−1^ and 7.7 % to 12.8 %, respectively, in the different subgroups. Bland–Altman plots of the estimated and measured VO_2_max (in L min^−1^ and mL kg^−1^ min^−1^) are given in Figs. 1 and 2, respectively. LoA for absolute values were −0.54 to 0.76 L min^−1^ in men and −0.61 to 0.44 L min^−1^ in women. The corresponding values for relative VO_2_max were −7.2 to 10.3 mL kg^−1^ min^−1^ in men, and −9.7 to 7.1 mL kg^−1^ min^−1^ in women, respectively. SEE for the absolute values were 0.33 L min^−1^ for men and 0.27 min^−1^ for woman, and the corresponding relative values were 4.17 and 4.26 mL kg^−1^ min^−1^, respectively.

### Comparison group

There were overall larger systematic errors and variations in estimations of VO_2_max by the EB-test_2012_ equation and the Åstrand method, compared with the EB-test_new_ equation (Table 4). Mean error was significantly lower for the EB-test_new_ compared with the EB-test_2012_ (*p* < 0.001). The adjusted *R*^2^ between estimated and measured values was significantly higher in EB-test_new_ compared with the Åstrand test. Compared with the new test equation, the larger coefficient of variation and SEE and lower *R*^2^ adjusted indicated a larger variability in the EB-test_2012_ and Åstrand test, respectively. In the full sample and in most subgroups, the difference between measured and estimated VO_2_max was lower for the new test equation compared with the other two tests.

In addition, difference between measured and estimated VO_2_max for the EB-test_new_ and the EB-test_2012_ equation was correlated with measured VO_2_max level in the comparison group. The associations between estimation error and VO_2_max level for the EB-test_2012_ were *ρ* = 0.37 in the full sample, and *ρ* = 0.44 and *ρ* = 0.83 (all *p* < 0.01) for women and men, respectively. For the EB-test_new_, these correlations were lower and only significant in woman; the corresponding values were *ρ* = 0.074 (*p* = 0.53) in the full sample, and *ρ* = 0.43 (*p* = 0.004) and *ρ* = 0.36 (*p* = 0.069) in women and men, respectively.

## Discussion

In the present paper, we have further developed a test, the EB-test presented in 2012, for the estimation of VO_2_max from submaximal work rates on cycle ergometers by including sex-specific prediction equations and expanding the test’s validity in respect to both VO_2_max and age range. Compared with the internal validation for the EB-test_2012_ (9.3 % and 0.30 L min^−1^), the variation between estimated and measured VO_2_max and corresponding SEE was lower for the EB-test_new_; 8.7 % and 0.28 L min^−1^. Cross validation in an external sample showed similar improved results. Subgroup analyses in both the internal validation sample and the cross-validation sample showed consistency in accuracy and precision between sexes, age-groups, and quartiles of VO_2_max. The correlation between the estimation error and VO_2_max level seen for the EB-test_2012_ was lower for the EB-test_new_ in the full sample and especially among men. This reduction may be partly explained by the inclusion of two new variables in the prediction equation, ΔPO variable, and HR at standard work rate.

The objective of the EB-test_new_, just as with the EB-test_2012_, is to provide a simple, time-effective and low-risk way to enable estimation of VO_2_max in settings outside the physiological laboratory. With the increased valid range for the test, it is now applicable on healthy individuals with wide ranging physical performance capacity. Another advantage of the EB-test_new_ is the further improved precision of the estimated VO_2_max compared with measured VO_2_max, with low variation between sexes and different age and VO_2_max groups (see Table 4). For example, by applying the variation between measured and estimated VO_2_max reported in the model group (8.4 % for men and 9.2 % for women), 95 out of 100 male individuals performing the EB-test_new_ with an actual VO_2_max of 3.0 L min^−1^ will be predicted within ±0.49 L min^−1^ and females within ± 0.54 L min^−1^. For a male or female with an absolute VO_2_max of 2.0 L min^−1^, the corresponding estimated VO_2_max is within 0.33 and 0.36 L min^−1^, respectively. However, these results from the EB-test_new_ rely on that the data for calculation are obtained in line with the description for test method and with the correct equipment (i.e., cycle ergometer), as described earlier. The same test manual and protocol should be applied for the EB-test_new_ as for the EB-test_2012_ (Ekblom-Bak et al. 2014), which is already in use. It is only the prediction equation that has been updated. The new equation is sex specific and with a wider age and VO_2_max ranges, with the inclusion of two additional test variables (the ΔPO variable and HR at the standard work rate) to reduce the estimation error and increase the precision of the estimation of VO_2_max.

There are a number of already existing submaximal tests for estimation of VO_2_max, where the participant performs an amount of submaximal work in the form of a step-up exercise, walking, running, or pedalling on a cycle ergometer (Brouha et al. 1943; Åstrand and Ryhming 1954; McArdle et al. 1972; Legge and Bannister 1986; Kline et al. 1987; Golding et al. 1989; Hartung et al. 1993; Swank et al. 2001; Solway et al. 2001; Bennett et al. 2015). Compared with previously described methods, the EB-test_new_ test has higher R^2^ than many step tests, which range from 0.22 to 0.90 (Perroni et al. 2013; Åstrand and Ryhming 1954; Santo and Golding 2003; McArdle et al. 1972; Francis and Culpepper 1989; Knight et al. 2014; Chatterjee et al. 2004). Compared with other studies on the validity of the Åstrand test, the EB-test_new_ test has similar *R*^2^ of 0.90 (Hartung et al. 1993). Validity is slightly higher for maximal treadmill running tests for the determination of VO_2_max, e.g., Balke test protocol, *R*^2^ 0.85 or Bruce test protocol, *R*^2^ 0.77 (Pollock et al. 1976). However, maximal tests include an all-out performance, in which VO_2_max is only one part. Furthermore, maximal running tests are not applicable in many situations, such as when testing older people, patients with orthopedic diagnoses, obesity or people unaccustomed to intense running. Furthermore, relative estimates or agreement, such as the correlation coefficient, are highly dependent on absolute range. In this present paper, we have the same absolute range in VO_2_max, making *R*^2^ comparisons meaningful. In the comparison group, the adjusted *R*^2^ for EB-test_new_ was 0.83, and the corresponding value for the Åstrand test method was 0.50.

Absolute measures of agreement indicate similar or slightly better validity for the EB-test_new_ (SEE: 4.2 mL kg^−1^ min^−1^ for men and 4.3 mL kg^−1^ min^−1^ for women, respectively) compared with other tests. The previous studies report an SEE of 8.9 mL kg^−1^ min^−1^ for the submaximal YMCA cycle ergometer test (Beekley et al. 2004) and for the Åstrand test method SEE from 4.3 mL kg^−1^ min^−1^ (Hartung et al. 1993) to 5.7 mL kg^−1^ min^−1^ (Cink and Thomas 1981). In the comparison group in the present paper, SEE for relative values based on the Åstrand test method was 5.6 mL kg^−1^ min^−1^, compared with the EB-test_new_ 4.1 mL kg^−1^ min^−1^ (data not shown).

The commonly found low accuracy and precision with the run- walk and step tests could be due to the inter-individual variance in morphology, body mass, gait, and mechanical efficiency, which may be induced when individuals perform these types of weight-bearing activities. The use of the cycle ergometers for submaximal testing diminishes some of the mechanical variations in performance, as the previous research has shown rather constant mechanical efficiency in mixed populations (Åstrand and Rodahl 1970).

Another factor influencing the accuracy of submaximal tests is the use of age-predicted maximal HR in the calculations, a postulation where there is large individual variations (Engels et al. 1998). For example, the Åstrand test uses a one-point work rate methodology combined with the above-mentioned assumption regarding maximal HR, which partly explains some of the measurement error (in the present study population *r* = 0.71 and CV 18.1 %, respectively). In the present test, there are no calculations of maximal HR; therefore, this potential source of error is eliminated. We believe that the inclusion of the ΔPO variable and HR at the low standard work rate in the EB-test_new_ equation explains some of the reduced variation in the low and high ends of the VO_2_max range, resulting in a higher precision throughout the valid VO_2_max span.

Although showing better accuracy and precision compared with the EB-test_2012_, there is still an estimation error left in the EB-test_new_ equation with increased VO_2_max level, albeit non-significant. One possible source of error could be the usage of the same and rather low standard work rate (≈30 W) in a population with wide ranging physical performance capacity. This rate of work may be too low for obtaining full stroke volume and thereby contributes to a variation in HR response at the standard work rate, especially in highly trained subjects (Blomqvist and Saltin 1983). To analyse this, we examined the correlation of percentage of VO_2_max on the standard work rate and measured VO_2_ during submaximal cycling (*n* = 110) and the estimation error. The correlation was *ρ* = 0.18 (*p* = 0.061). The percentage of VO_2_max on the standard work rate was therefore ruled out as a main source of the estimation error. However, other factors, such as blood flow distribution and blood pressure, related to high VO_2_max level, may influence the estimation error.

Another potential source of error that may influence on the precision of the submaximal test is the reliance on the existence of a linear relationship between VO_2_ and power output, as previously shown by Åstrand and Rodahl (Åstrand and Rodahl 1970). This notion has been questioned due to later findings of a non-linear relation between VO_2_ and power output (Zoladz et al. 1995). This non-linearity may affect the ability of a submaximal test to predict VO_2_max. However, the non-linearity of this relationship seems to be found at high intensities above the anaerobic threshold (Zoladz et al. 1998; Majerczak et al. 2012). Nonetheless, the issue of non-linearity may well be an important factor for the proper execution of the test, stressing the importance of not choosing an individual work rate that is too high.

### Strengths and limitations

A strength of the EB-test_new_ equation is the consistent high accuracy and precision in the external cross-validation sample, as well as in the different subgroups (men and women, different age-groups and a wide range of VO_2_max levels). This is of particular interest with regard to the fact that the new equation is based on a relatively large and heterogeneous sample, with a wide variation in age and VO_2_max. We, therefore, believe that the test now is suitable for most non-diseased individuals. The use of sex specific prediction equations, rather than controlling for sex within an equation, has undoubtedly contributed to better precision.

A common issue in studies involving maximal testing is the selection bias, often including more fit individuals than in the general population. This may limit the accuracy of the prediction equation in the general population. One way to analyse the representativeness of the present model group for the general population (with special regard to age), is to compare the decrease in VO_2_max with age between the model group and previously reported values from general population samples. The age-related decline per decade in measured VO_2_max for the model group sample in this study was −9.1 % per 10-year, compared with -6.5 % in 10,973 men and women, where VO_2_max was obtained from maximal testing on cycle ergometer (Eriksen et al. 2015) and −6.9 % in 3678 men and women, where VO_2_max was obtained from treadmill testing (Loe et al. 2013). The difference in slope may be due to a higher mean fitness among the young participants in the present paper, and may thus express a limited degree of bias. The implication of this bias on the validity of the test may be regarded as low.

Moreover, a submaximal test only estimates VO_2_max based on variables obtained during the submaximal exercise. Hence, individuals who deviate in physiological characteristics from the individuals included in the model group, for example with an extremely high or low cardiorespiratory fitness, exceptional work efficiency or abnormal HR response, may obtain an estimated VO_2_max further from their actual VO_2_max than expected. Furthermore, any medications that may have an influence on HR, also may affect the results from the test. This prediction equation is based on ∆HR, and the ∆HR relation to power output, whereas medications, such as beta block, may alter these relationships. Future research should focus on the feasibility of the EB test in clinical populations. Another scope of interest is the ability of the EB test to detect a change in actual VO_2_max over time, for example as a consequence of a training intervention. The use of the test in this situation has not yet been evaluated. To date, the recommendation is to keep the same high individually chosen work rate when monitoring an individual over time.

## Conclusion

In the present paper, we have further developed an easy administered, non-expensive, and accurate submaximal ergometer test for the estimation of VO_2_max. The EB-test_new_ estimates VO_2_max throughout a wide range of ages and fitness levels, and can be used in health screenings and in research studies in large populations and in the general population.

## Abbreviations

bpm: Beats per minute
EB-test_2012_: The Ekblom-Bak test with the original prediction equation (Epub 2012 Nov 6)
EB-test_new_: The Ekblom Bak-test with the new prediction equation
HR: Heart rate
RPE: Rate of perceived exertion
rpm: Revolutions per minute
VO_2_: Oxygen uptake
VO_2_max: Maximal oxygen uptake
ΔHR: The difference in HR between the high and low work rate
ΔPO: The difference in work rate between the high and low work rate
